# Male Inmate Profiles and Their Biological Correlates

**DOI:** 10.1177/070674371405900807

**Published:** 2014-08

**Authors:** Mathilde Horn, Stephane Potvin, Jean-François Allaire, Gilles Côté, Gabriella Gobbi, Karim Benkirane, Jeanne Vachon, Alexandre Dumais

**Affiliations:** 1 Researcher, Philippe-Pinel Institute of Montreal, Mental Health University Institute of Montreal, Department of Psychiatry, University of Montreal, Montreal, Quebec; Psychiatrist and Researcher, University Medical Centre, Functional Neuroscience and Disorders Laboratory, Lille North of France University, Lille, France.; 2 Researcher, Mental Health University Institute of Montreal, Department of Psychiatry, University of Montreal, Montreal, Quebec.; 3 Statistician, Philippe-Pinel Institute of Montreal, Montreal, Quebec.; 4 Director, Research Centre, Philippe-Pinel Institute of Montreal, Montreal, Quebec; Professor, Department of Psychology, University of Quebec at Trois-Rivières, Trois-Rivières, Quebec.; 5 Associate Professor, Department of Psychiatry, McGill University and McGill University Health Centre, Montreal, Quebec.; 6 Clinical Biochemist, Maisonneuve-Rosemont Hospital, Biochemistry Laboratory, Department of Biochemistry, University of Montreal, Montreal, Quebec.; 7 Research Coordinator, Philippe-Pinel Institute of Montreal, Montreal, Quebec.; 8 Psychiatrist and Researcher, Philippe-Pinel Institute of Montreal, Mental Health University Institute of Montreal, Department of Psychiatry, University of Montreal, Montreal, Quebec.

**Keywords:** personality disorders, impulsivity, substance use disorders, testosterone, cortisol, clustering

## Abstract

**Objective:**

Borderline and antisocial personality disorders (PDs) share common clinical features (impulsivity, aggressiveness, substance use disorders [SUDs], and suicidal behaviours) that are greatly overrepresented in prison populations. These disorders have been associated biologically with testosterone and cortisol levels. However, the associations are ambiguous and the subject of controversy, perhaps because these heterogeneous disorders have been addressed as unitary constructs. A consideration of profiles of people, rather than of exclusive diagnoses, might yield clearer relationships.

**Methods:**

In our study, multiple correspondence analysis and cluster analysis were employed to identify subgroups among 545 newly convicted inmates. The groups were then compared in terms of clinical features and biological markers, including levels of cortisol, testosterone, estradiol, progesterone, and sulfoconjugated dehydroepiandrosterone (DHEA-S).

**Results:**

Four clusters with differing psychiatric, criminal, and biological profiles emerged. Clinically, one group had intermediate scores for each of the tested clinical features. Another group comprised people with little comorbidity. Two others displayed severe impulsivity, PD, and SUD. Biologically, cortisol levels were lowest in the last 2 groups and highest in the group with less comorbidity. In keeping with previous findings reported in the literature, testosterone was higher in a younger population with severe psychiatric symptoms. However, some apparently comparable behavioural outcomes were found to be related to distinct biological profiles. No differences were observed for estradiol, progesterone, or DHEA-S levels.

**Conclusions:**

The results not only confirm the importance of biological markers in the study of personality features but also demonstrate the need to consider the role of comorbidities and steroid coregulation.

## Abstract (fr)

**Objectif:**

Les troubles de la personnalité (TP) limite et antisociale ont des caractéristiques cliniques communes (impulsivité, agressivité, troubles liés à l’utilisation de substances [TUS], et comportements suicidaires) qui sont beaucoup surreprésentées dans les populations carcérales. Ces troubles ont été associés biologiquement aux taux de testostérone et de cortisol. Cependant, les associations sont ambiguës et font l’objet de controverses, peut-être parce que ces troubles hétérogènes ont été abordés comme des construits unitaires. Un examen des profils des personnes, plutôt que des diagnostics exclusifs, pourrait produire des relations plus nettes.

**Méthodes:**

Dans notre étude, nous avons employé l’analyse des correspondances multiples et l’analyse typologique pour identifier les sous-groupes parmi 545 détenus récemment condamnés. Les groupes ont ensuite été comparés en ce qui concerne les caractéristiques cliniques et les marqueurs biologiques, notamment les taux de cortisol, de testostérone, d’estradiol, de progestérone, et de déhydroépiandrostérone sulfoconjuguée (DHEA-S).

**Résultats:**

Quatre groupes ayant des profils psychiatriques, criminels, et biologiques différents se sont démarqués. Cliniquement, un groupe avait des scores intermédiaires pour chacune des caractéristiques cliniques testées. Un autre groupe comportait des personnes ayant peu de comorbidité. Deux autres présentaient une impulsivité grave, un TP et un TUS. Biologiquement, les taux de cortisol étaient les plus faibles dans les 2 derniers groupes, et les plus élevés dans le groupe ayant peu de comorbidité. Conformément aux résultats précédents rapportés dans la littérature, la testostérone était plus élevée dans une population plus jeune présentant de graves symptômes psychiatriques. Toutefois, des résultats comportementaux apparemment comparables se sont révélés être reliés à des profils biologiques distincts. Aucune différence n’a été observée pour l’estradiol, la progestérone ou les taux de de DHEA-S.

**Conclusions:**

Non seulement les résultats confirment l’importance des marqueurs biologiques dans l’étude des traits de personnalité, mais ils démontrent aussi la nécessité d’examiner le rôle des comorbidités et de la co-régulation des stéroïdes.

In the past few years, many studies have reported on the overrepresentation of people with mental disorders in the criminal justice system.^1^–^5^ BPDs and ASPDs have been found to affect large proportions of offenders (25% of females and 47% of males) and play a significant role in the occurrence of violent crimes.^6^–^10^ These 2 PDs share several comorbidities, including SUDs,^11^–^17^ impulsivity,^11^,^13^,^18^–^21^ and aggressiveness.^7^,^22^–^24^ Moreover, patients with BPD and ASPD are particularly at risk of self-inflicted injury or suicide attempts^6^,^21^; these risks increase with impulsivity^6^,^19^,^25^,^26^ and SUD.^14^

Studies have found all these criminogenic clinical features to be related to specific biological—neuroendocrine hormone—profiles. Thus research has suggested that cortisol levels are related to particular clinical features,^27^ but the findings have been conflicting.^28^,^29^ For example, while cortisol has been associated with SUDs^30^ and aggressiveness,^31^ the associations have been largely inconsistent.^28^,^29^ Suicidal behaviours have also been linked to high levels of cortisol,^32^,^33^ whereas impulsivity has been linked to low levels. Finally, in patients with ASPD, cortisol may be differentially involved depending on the type of antisocial behaviour and associated comorbidities.^28^

Testosterone, too, has been found to be a factor in aggression^31^,^34^–^36^ and antisocial behaviour,^35^,^37^ but no one-to-one relation between higher testosterone levels and aggression has been demonstrated.^29^ According to some authors, testosterone may be more closely related to dominant behaviour than to aggressiveness.^36^ Associations have also been found between testosterone levels and risk of suicide attempts,^32^,^38^,^39^ but again the findings have been contradictory.

Further, research has suggested an association between estradiol and certain clinical disorders and symptoms observed in inmates, such as aggressiveness,^29^,^37^ SUD,^40^ and BPD.^41^ However, few studies have explored the link, and the results have been inconsistent.

Testosterone and estradiol are derived from DHEA, which can be interconverted with its sulfoconjugated derivative, DHEA-S.^42^ DHEA has been found to be involved in aggressiveness,^29^ SUD,^43^ and suicidal behaviour,^44^ but its precise function remains unclear.^45^,^46^

**Clinical Implications**

- Some apparently comparable behavioural outcomes may be associated with different biological processes.
- Cortisol, testosterone, and the ratio between them were related to specific personality features but estradiol, progesterone, and DHEA-S were not.
- The identification of clinical profiles associated with criminal behaviour should help optimize treatment.

**Limitations**

- This is a correlational, cross-sectional study.
- Biological markers were compared in a smaller group of participants than the clinical and criminal features.

To our knowledge, no link has been found between progesterone and psychopathological features, though various authors suggest the hormone plays a role in the regulation of brain activity through the modulation of neurotransmitter synthesis and release.^47^

Strong interactions between testosterone and cortisol have led researchers to consider the testosterone–cortisol ratio as more relevant than the absolute concentrations of the hormones.^46^ For example, it has been suggested that correlations between cortisol and aggression may be explained by the ability of cortisol to lower testosterone levels.^29^ Most of the studies considering the testosterone–cortisol ratio have shown it to be linked to socially aggressive behaviour.^31^,^48^

While these neuroendocrine hormones and offender disorders or behaviours thus seem to be related, the associations remain unclear.^28^,^29^ The lack of clarity may be due to heterogeneous disorders being addressed as unitary constructs.^1^,^49^ Considering peoples’ profiles in terms of PDs, impulsivity, aggression, SUDs, and suicidal behaviours rather than of exclusive diagnoses may result in a better understanding of the association between biological markers and clinical features. In our study, we therefore sought to elicit the subjects’ different clinical profiles to compare them with respect to their biological markers. We decided to conduct an MCA, an approach that makes it possible to analyze patterns of relations between several variables and determine clusters of subjects with common clinical features.^50^–^53^ We then compared these clusters in terms of criminal and psychiatric history and biological profiles.

## Methods

Our study is part of a larger project to describe mental disorders (such as psychotic disorders, mood disorders, anxiety disorders, SUDs, and PDs) and intellectual disability in newly convicted inmates in a federal prison setting in Quebec. Offenders admitted to the Quebec RRC, a facility responsible for assessing offenders condemned to a sentence of 2 years or more, were approached. Recruitment took place between October 2007 and November 2011. Data were obtained through standardized interviews, consultation of prison records, and laboratory tests. The project was approved by local ethics committees: Comité d’éthique de la recherche de l’Université du Québec à Trois-Rivières (CER-07–122–07.04), Comité d’éthique de la recherche de l’Institut Philippe-Pinel de Montréal (070216/E/E/A/6), and McGill University Institutional Review Board (A02-B05–08A).

### Participants

For the larger project, it was estimated that a sample of 574 participants would be needed (in accordance with the prevalence of mental disorders in this population). Given the annual intake of new inmates in the Quebec RRC and refusals or interruptions before the end of the study, it was calculated that during 3 years, one-quarter of the new inmates had to be approached to take part. One in 4 from the list of new admissions was thus contacted. Among the 731 offenders approached, 579 (79.2%) agreed to participate; 545 (74.5%) completed the entire interview; and 368 (50.3%) consented to the blood test. All of the subjects gave their written informed consent to the study after receiving a detailed description of the research protocol. The study population was made up of French- or English-speaking males aged 18 to 84 years (mean 39 years, SD 13).

### Measures

All questionnaires were administered by trained psychologists and graduate psychology students.

## Clinical and Criminal Histories

Demographic data, psychiatric history (previous psychiatric consultations and hospitalizations), and criminal history (incarcerations in the last year, self-reported undetected offences, and security level of the detention establishment) were acquired from interviews and complemented by consultation of the medical and criminal records.

### Diagnoses

PD diagnoses were determined by the SCID Axis II Disorders.^54^ Assessments of lifetime SUDs were based on the SCID Axis I Disorders.^55^

### Impulsivity

The Barratt Impulsiveness Scale,^56^ a 30-item self-report questionnaire, was used to assess impulsivity levels. This scale measures 3 components of impulsivity: motor impulsiveness, defined as a tendency to act on the spur of the moment; attentional impulsiveness, characterized by lack of focus on the task at hand; and nonplanning impulsiveness, described as lack of a sense of the future.^49^

### Aggressive or Violent Behaviour

Past aggressive or violent behaviour was evaluated by the MacArthur Community Violence Instrument.^57^,^58^ This self-report measure was chosen because it is an appropriate tool for the assessment of past history of violence and provides information on the occurrence and number of participant-perpetrated aggressive events. Severity of aggression was classified using a 2-level scale: severe aggressive behaviour was defined as murder or attempted murder, threat using a weapon, sexual assault, or any other violence with injury to a victim; minor or general aggressive behaviour comprised acts without the use of a weapon or without injury.

### Self-Harm and Suicidal Behaviours

Self-injury was evaluated with the Lethality of Suicide Attempt Rating Scale, which measures the lethality of suicide attempts on a scale of 1 to 10 by the severity of the method used and the circumstances in which the event took place.^59^ For our study, we extracted the number of self-harm or suicidal behaviours.

### Steroid Hormones

Steroid hormone levels were analyzed using the blood samples of consenting participants who gave their sample before 9:00 AM (*n* = 158). To minimize the effect of variations in steroid levels during the course of the day,^60^,^61^ participants whose blood samples were collected after 9:00 AM were excluded from this analysis. However, to assess the comparability of the subsample with the total sample, the distributions of the clusters in both the larger and smaller groupings were checked for equivalence (see Data Analysis, Cluster Comparisons).

Specimens from all participants who underwent a venous blood draw (*n* = 368) were collected in 6 mL BD Vacutainer gel tubes (BD Canada, Mississauga, ON). After being spun at a speed of 3200 rpm for 15 minutes, serums were extracted and stored at −80 °C. All specimens were analyzed for cortisol and testosterone by chemiluminescence immunoassay method on an ARCHITECT i2000 analyzer (Abbott Diagnostic, Mississauga, ON). All assays used 25 μL of serum with a lower detection limit of 22.1 nmol/L (cortisol), 0.28 nmol/L (testosterone), 36.7 pmol/L (estradiol), 0.32 nmol/L (progesterone), and 0.08 μmol/L (DHEA-S).

### Data Analysis

#### Multiple Correspondence Analysis

MCA is an extension of simple correspondence analysis designed to analyze relations between variables represented in a 2-way frequency cross-tabulation table.^62^ It is an exploratory graphical technique that allows one to identify individual profiles using the variables included in the analysis. The rows and columns of the table are assumed to be points in a high-dimensional Euclidean space. Associations are ascertained by calculating distances between points in the space, that is, the chi-square distances between people in different categories of the variables under study. The aim is to redefine the principal dimensions or axes of the space to capture most of the inertia (which may be interpreted as the explained variance or *R*^2^). The output of MCA provides eigenvalues plotted by increasing dimensionality and resulting in a falling curve. The number of dimensions is determined by the point at which the curve bends and flattens out (the elbow) after sloping relatively steeply downward.^63^ A scree test was also applied to determine how many dimensions to retain.^64^

#### Cluster Analysis

A hierarchical clustering technique applied to a limited number of the dimensions obtained from the MCA has been shown to be more efficient for classifying cases.^62^ Ward’s criterion, which results in minimum loss of inertia, was used as an agglomerative method. To determine the number of clusters to retain, we used the dendrogram (aggregation tree) that emerged from the hierarchical clustering to establish the number of classes. To describe the specific characteristics of each profile, we used the value test.^65^ A cut-off of 2 (*P* < 0.05) was chosen; this cut-off is for descriptive purposes only and should not be interpreted as a hypothesis test as all the variables were used to create the clusters.

#### Cluster Comparisons

Comparisons between clusters were based on psychiatric and criminal history with variables not used in the first analysis and on biological profiles (testosterone, cortisol, testosterone–cortisol ratio, estradiol, progesterone, and DHEA-S). Data were recoded into dichotomized variables (greater or less than the median value) to characterize the clusters in terms of psychiatric and criminal profiles. Comparisons were then completed with chi-square tests. Given the impact of age on neurosteroid levels, the analyses involving the biological comparisons were run by age group. Four age groupings were established, and, within each of these, medians were used to dichotomize low and high concentrations of the biological markers. Cluster comparisons were performed with chi-square tests as well. These analyses were conducted using Statistical Analysis Software for Windows, version 9.1 (SAS Institute Inc, Cary, NC). Statistical power analyses were performed using Power and Precision 4 (Biostat, Englewood, NJ).^66^

## Results

### MCA Dimensions

As participants for whom data were missing were excluded, the first analyses involved 545 subjects. The MCA revealed 3 dimensions, explaining more than 99% of the Benzécri adjusted inertia.^67^ Although Benzécri’s correction formula provides a better estimate of inertia, its known tendency to yield optimistic results^50^ would seem to account for the high proportion of explained inertia here. The first dimension, accounting for 91.5% of the inertia, separates impulsive people with ASPD who use cannabis, cocaine, and other drugs (that is, drugs other than cannabis, cocaine, sedatives, and stimulants) from the other people. The second dimension, which explains 6.5% of the inertia, comprises people with BPD features and history of self-harm or suicidal behaviour. The third dimension explains 1.1% of the inertia and differentiates aggressive people with unstable relationships and sudden mood change from nonaggressive people presenting nonplanning and cognitive impulsivity and using cocaine, sedatives, and other drugs.

### Cluster Analysis

Cluster analysis partitioned the 545 subjects into 4 mutually exclusive clusters, as presented in online eTable 1. Cluster 3 appears to reflect the complete study sample, with clinical features in comparable proportions. Cluster 1 comprises all people without any PD and presenting very few other comorbidities (impulsivity, aggressiveness, SUD, and self-harm). The 2 other clusters comprise people with severe disorders. Cluster 2 is characterized mainly by polysubstance users with high levels of impulsivity and aggressiveness and ASPD traits. Cluster 4 groups together all people with a diagnosis of BPD, ASPD, and BPD traits, a high level of impulsivity, suicidal behaviours, and SUD marked by an overrepresentation of sedative use.

### Cluster Comparisons

We evaluated the validity of our 4-cluster solution by comparing the clusters in terms of sociodemographic, criminal, and psychiatric characteristics. The results are summarized in Table 2. The 4 groups differ significantly in each of these characteristics. As previously noted, cluster 3 has features in proportions comparable with those in the sample as a whole. Cluster 1 is characterized by older people (median age 45.0 years, SD 14.7) with higher levels of education, little previous psychiatric history, and few prior judicial interventions. In contrast, cluster 4 comprises younger people (median age 33.5 years, SD 10.4) with many criminal characteristics, the greatest number of separations in their romantic relationships, and the most extensive psychiatric history as measured by previous psychiatric consultations and hospitalizations. Cluster 2 also comprised young people (median age 33.0 years, SD 11.0) with a low level of education, several previous psychiatric consultations and hospitalizations, and the most extensive criminal history.

The 4 clusters were compared in terms of concentrations of cortisol, testosterone, estradiol, progesterone, DHEA-S, and the testosterone–cortisol ratio. The chi-square goodness-of-fit test, which compares observed and expected frequencies in each category to test whether all categories contain the same or a specific proportion of values, was applied to check the equivalence of the distributions between the total and smaller samples. The distribution of the clusters within this subgroup was found to be equivalent to that in the total sample (χ^2^ = 1.14, *df* = 3, *P* = 0.77). The sociodemographic, criminal, and psychiatric characteristics of the initial total sample and the subsample used for biological analyses were also found to be comparable, confirming that the subsample is representative of the study population as a whole (details of the sociodemographic, criminal, and psychiatric characteristics of the subsample are available on request). Table 3 shows significant differences between the clusters in terms of testosterone levels and the testosterone– cortisol ratios. The highest testosterone concentrations and testosterone–cortisol ratios are in cluster 4; the lowest are in cluster 2. Cluster 1 is associated with a high testosterone level and a moderate testosterone–cortisol ratio. Cluster 3 displays intermediate values. A nonsignificant difference was also found for cortisol levels, with the lowest concentrations in clusters 2 and 4 and the highest in cluster 1. Again, cluster 3 displays intermediate levels. No differences are observed for estradiol, progesterone, and DHEA-S.

## Discussion

In our study, we sought to elicit profiles of newly sentenced offenders based on clinical features typically associated with this population and to relate them to biological characteristics. Using MCA and cluster analysis, we identified 4 clusters distinguished by clinical traits that also differ significantly in terms of sociodemographic, criminal, and psychiatric characteristics.

Cluster 3 is the intermediate cluster. The prevalence of clinical features and criminal characteristics appears to be comparable with what has been observed in other studies of male inmates.^68^,^69^ Indeed, people with ASPD; impulsivity; history of severe aggressions; and abuse of, or dependence on, alcohol, cannabis, and cocaine account for only about one-half of this cluster, a proportion similar to that in the sample as a whole. Biological marker levels in this group are also similar to those in the greater study population.

Cluster 1 represents an older group with no PD. It comprises older people who, unlike subjects in the other groups, had few incarcerations in the last year, few offences without judicial intervention, relatively few psychiatric disorders, and, most particularly, no PD. It might be suggested that these differences are due solely to the more advanced age of people in this group as SUD,^70^ criminality,^29^ and cluster B PD^71^,^72^ have been consistently reported to decrease with age. Still, the small numbers of relationships and separations, psychiatric consultations and hospitalizations, and low criminal history scores strongly suggest that this group constitutes a distinct profile.

The group is characterized by high levels of testosterone and cortisol, and a medium testosterone–cortisol ratio. A high level of cortisol is consistent with the hypothesis regularly put forward of a negative association between cortisol and the severity of disorders in terms of impulsivity,^73^–^75^ aggressiveness, and antisocial behaviours.^29^,^31^,^48^,^73^ The high level of testosterone is more surprising and tends to support the notion that testosterone plays a role in dominant behaviour, rather than in aggressiveness. Further, it has been reported that the testosterone–aggression association is stronger among subjects with a low level of cortisol.^27^ Hence the interaction between high testosterone and low cortisol may account for aggressive behaviours better than testosterone alone. No specific clinical feature that could account for the risk of criminality emerged for this older group, and more research seems necessary to cast further light on the profile.

Clusters 2 and 4 are the groups with the most severe psychiatric and criminal characteristics. Both clusters are comprised of young people with ASPD features and numerous comorbidities, including impulsivity, aggressiveness, and SUD, psychiatric history of previous consultations and hospitalizations, and an extensive criminal history. In keeping with previous findings,^31^,^73^ both clusters are associated with low levels of cortisol. However, while cluster 4 displays high testosterone levels and a high testosterone–cortisol ratio, cluster 2 displays low scores on these measures, suggesting clinical and etiological differences between the groups.

Cluster 4, the severe borderline group, is notable for the high prevalence of BPD associated with a high frequency of suicidal behaviour. As expected, this cluster involves the more extensive psychiatric history (that is, it includes more people with a history of consultations and hospitalizations) known to obtain in BPD patients.^8^,^76^,^77^ This group is consequently characterized by the association of ASPD with BPD, a codiagnosis that has been found to be related to heightened risk of criminality.^1^,^7^ Biologically, the group is associated with the highest testosterone level and testosterone–cortisol ratio of the 4 clusters, thus supporting the association between a high testosterone–cortisol ratio and predisposition to impulsive aggression.^78^ Finally, this group is marked by a particularly high level of sedative abuse, which, in this context, may represent an emotion-regulation function^79^ or an equivalent of suicidal behaviour.

Cluster 2 emerges as a severely antisocial and criminal group, displaying the most severe ASPD, criminal features, and SUD. Unexpectedly, it presents the lowest testosterone levels and testosterone–cortisol ratios. Therefore, antisocial behaviour in cluster 2 may be related to different mechanisms than those suggested for cluster 4. Animal and human studies have reported decreased testosterone during chronic consumption of alcohol, cannabis, and cocaine.^29^,^80^–^82^ Such consumption has also been shown to contribute strongly to impulsivity,^12^,^14^,^15^,^19^,^83^ PD,^84^ and violent behaviour.^69^ Consequently, we postulate that antisocial behaviour in this group may arise from SUD. SUD is also known to promote criminal behaviour,^15^,^68^,^85^ thus accounting for the cluster’s criminal profile.

Comparable behavioural outcomes in clusters 2 and 4 may thus ultimately stem from different processes. Indeed, the differences in the biological profiles of clusters 2 and 4 strongly suggest different etiological mechanisms. Thus these findings confirm that associations between neuromodulators and antisocial behaviour can vary considerably depending on the nature of the antisocial behaviour.^28^

Although a robust methodology^50^,^53^ was applied to more than 500 people to elicit these profiles, some limitations should be noted. First, the research was conducted with male offenders only. Second, the biological marker comparisons involved fewer participants than the clinical and criminal comparisons and were run on a smaller group of people than the one used for the MCA. As a minimum number of data are required to conduct an MCA and obtain adequate results,^52^ the analysis was performed on the total sample, and the equivalence of the distributions between the total sample and the smaller one was verified. The results regarding testosterone, cortisol, and the ratio between them are particularly noteworthy given the smaller sample size; further analyses with larger groups of participants are recommended. The results also suggest that estradiol, progesterone, and DHEA-S levels are not comorbidities that characterize the groups, though the absence of any association with these biological markers may be due to the small number of subjects included in the analyses. To cast more light on this last point, it would be valuable to compare the concentration of biological markers in this offender population with that in a control group of people with no criminal history or psychiatric disorders. Finally, this is a correlational, cross-sectional study, which limits the possibility of determining causal relation between the variables.

## Conclusion

In our study, we sought to define profiles of offenders in terms of impulsivity, aggressiveness, and SUD, factors that contribute substantially to the risk of criminality. Four distinct groups emerged, differentiated by clinical features and biological profiles. These results confirmed the importance of cortisol and testosterone in relation to impulsivity and aggressiveness. However, they also demonstrated that the study of the association between behavioural and biological profiles necessitates a comprehensive approach, incorporating comorbidities and steroid coregulation. The identification of clinical profiles contributing to criminal behaviour should help optimize treatment. Specific treatments for the comorbidities associated with greater risk of criminal behaviour may help reduce the occurrence of offences.^15^,^49^ Biological support for the different clinical profiles may provide a basis for adapted medical therapies.

## 



## Figures and Tables

**Table 2:** Comparison of sociodemographic, criminal, and psychiatric characteristics

Variable	Total sample, % *n* = 545	Cluster 1, % (32.5%) *n* = 177	Cluster 2, % (26.6%) *n* = 145	Cluster 3, % (31.4%) *n* = 171	Cluster 4, % (9.5%) *n* = 52	χ^2^ (*df) P*
Age, years						47.6 (9) *<*0.001
≤30	31.6	23.7	40.7	31.0	34.6	
31–40	24.6	17.5	30.3	26.3	26.9	
41–50	25.7	26.6	19.3	28.1	32.7	
>50	18.2	32.2	9.7	14.6	5.8	
Education						35.5 (9) <0.001
No regular	0.6	1.7	0.0	0.0	0.0	
Primary or secondary	80.4	68.4	86.9	83.0	94.2	
Postsecondary	15.2	21.5	11.7	14.6	5.8	
University	3.9	8.5	1.4	2.3	0.0	
Relationships, median = 2						20.6 (3) <0.001
≤Median	60.4	74.0	53.1	55.0	51.9	
>Median	39.6	26.0	46.9	45.0	48.1	
Separations, median = 3						13.6 (3) 0.004
≤Median	64.4	74.6	59.3	62.0	51.9	
>Median	35.6	25.4	40.7	38.0	48.1	
Number of jobs, median = 5						15.6 (3) 0.001
≤Median	51.4	63.3	47.6	45.6	40.4	
>Median	48.6	36.7	52.4	54.4	59.6	
Previous psychiatric consultations						20.8 (3) <0.001
Yes	47.0	38.4	51.0	44.4	73.1	
No	53.0	61.6	49.0	55.6	26.9	
Previous psychiatric hospitalizations						44.9 (3) *<*0.001
Yes	17.1	10.2	20.0	12.3	48.1	
No	82.9	89.8	80.0	87.7	51.9	
Incarceration during the last year						56.4 (3) <0.001
Yes	29.0	11.3	49.0	28.2	36.5	
No	71.0	88.7	51.0	71.8	63.5	
Other offences without judicial intervention						128.8 (3) <0.001
Yes	53.3	19.3	77.9	62.0	71.2	
No	46.7	80.7	22.1	38.0	28.8	
Level of security of the establishment						55.2 (6) <0.001
Minimum	34.1	52.6	20.0	30.4	23.1	
Medium	59.7	45.1	67.6	66.1	65.4	
Maximum	6.3	2.3	12.4	3.5	11.5	

**Table 3:** Comparison of biological markers

Variable	Total sample, % *n* = 158	Cluster 1, % (33.5%) *n* = 53	Cluster 2, % (24.7%) *n* = 39	Cluster 3, % (28.5%) *n* = 45	Cluster 4, % (13.3%) *n* = 21	χ^2^ (*df) P*	Effect size	Power (1 –β)
Cortisol						7.29 (3) 0.06	0.21	0.61
≤Median	51.6	36.7	60.5	55.8	65.0			
>Median	48.4	63.3	39.5	44.2	35.0			
Testosterone						7.95 (3) <0.05	0.23	0.66
≤Median	51.3	44.0	68.4	55.8	35.0			
>Median	48.7	56.0	31.6	44.2	65.0			
Estradiol						1.04 (3) >0.10	0.09	0.14
≤Median	53.7	55.1	47.1	53.5	61.1			
>Median	46.3	44.9	52.9	46.5	38.9			
DHEA-S						4.09 (3) >0.10	0.16	0.36
≤Median	53.8	53.8	44.7	65.1	45.0			
>Median	46.2	46.2	55.3	34.9	55.0			
Progesterone						1.35 (3) >0.10	0.09	0.14
≤Median	58.4	52.0	63.9	57.5	65.0			
>Median	41.6	48.0	36.1	42.5	35.0			
T-C ratio						9.55 (3) <0.05	0.25	0.76
≤Median	50.3	53.1	65.8	44.2	25.0			
>Median	49.7	46.9	34.2	55.8	75.0			

DHEA-S = sulfoconjugated dehydroepiandrosterone; T-C = testosterone–cortisol

## Abbreviations

ASPD: antisocial personality disorder
BPD: borderline personality disorder
DHEA: dehydroepiandrosterone
DHEA-S: sulfoconjugated DHEA
MCA: multiple correspondence analysis
PD: personality disorder
RRC: Regional Reception Centre
SCID: Structured Clinical Interview for the Diagnostic and Statistical Manual or Mental Disorders, Fourth Edition
SUD: substance use disorder
