# Supplementary material for: Male Inmate Profiles and Their Biological Correlates
Source: Can J Psychiatry. 2014 Aug;59(8):441–9. doi: 10.1177/070674371405900807 (PMC4143301; doi:10.1177/070674371405900807)
Supplement: Supplementary file 1 [file CJP-2014-vol59-August-441-449-eTable1.pdf]

Horn M, Potvin S, Allaire J-F, et al. Male inmate profiles and their biological correlates. Can J Psychiatry. 2014;59(8):441–449.

Table 1: Associations of the different profiles of individuals with the study variables

**Variables**	**Total sample** n = 545	**Cluster 1** (32.5%) n = 177	**Cluster 2** (26.6%) n = 145	**Cluster 3** (31.4%) n = 171	**Cluster 4** (9.5%) n = 52
**ASPD**					
Diagnosis completed					
Present	45.7%	*0%*	**83.4%**	**52.6%**	**73.1%**
Absent	54.3%	**100%**	*16.6%*	*47.4%*	*26.9%*
Failure to conform to social norms					
Present	81.4%	*49.7%*	**100%**	**94.7%**	**94.2%**
Absent	18.6%	**50.3%**	*0%*	*5.3%*	*5.8%*
Irritability and aggressiveness					
Present	42.8%	*8.5%*	**64.1%**	**56.7%**	53.8%
Absent	57.2%	**91.5%**	*35.9%*	*43.3%*	46.2%
Impulsivity					
Present	38.8%	*6.2%*	**64.8%**	39.8%	**73.1%**
Absent	61.2%	**93.8%**	*35.2%*	60.2%	*26.9%*
Reckless disregard for safety					
Present	54.9%	*24.3%*	**77.2%**	**62.0%**	**73.1%**
Absent	45.1%	**75.7%**	*22.8%*	*38.0%*	*26.9%*
Deceitfulness					
Present	33.8%	*8.5%*	**55.2%**	37.4%	**48.1%**
Absent	66.2%	**91.5%**	*44.8%*	62.6%	*51.9%*
Consistent irresponsibility					
Present	27.9%	*4.5%*	**57.9%**	*22.2%*	**42.3%**
Absent	72.1%	**95.5%**	*42.1%*	**77.7%**	*57.7%*
Lack of remorse					
Present	58.2%	*33.9%*	**71.7%**	**70.2%**	63.5%
Absent	41.8%	**66.1%**	*22.3%*	*29.8%*	36.5%
**BPD**					
Diagnosis completed					
Present	7.2%	*0%*	*0%*	*0%*	**75.0%**
Absent	92.8%	**100%**	**100%**	**100%**	*25.0%*
Impulsivity					
Present	52.8%	*8.5%*	**89.0%**	55.6%	**94.2%**
Absent	47.1%	**91.5%**	*11.0%*	44.4%	*5.8%*
Intense and inappropriate anger					
Present	25.7%	*4.5%*	**36.6%**	27.5%	**61.5%**
Absent	74.3%	**95.5%**	*63.4%*	72.5%	*38.5%*
Chronic feeling of emptiness					
Present	18.5%	*5.1%*	**24.8%**	*8.8%*	**78.5%**
Absent	81.6%	**94.9%**	*75.2%*	**91.2%**	*21.2%*
Unstable and intense interpersonal relationships	12.5%	*2.3%*	16.6%	*7.6%*	**51.9%**
Present	87.5%	**97.7%**	83.4%	**92.4%**	*48.1%*

	Absent					
Recurrent suicidal behaviour		11.4%	*1.7%*	13.8%	*1.2%*	**71.2%**
	Present	88.6%	**98.3%**	86.2%	**98.8%**	*28.8%*
	Absent					
Affective instability		10.7%	*1.7%*	11.7%	*6.4%*	**51.9%**
	Present	89.3%	**98.3%**	99	**93.6%**	*48.1%*
	Absent					
Identity disturbance		7.7%	*0%*	**13.1%**	*2.9%*	**34.6%**
	Present	92.3%	**100%**	86.9%	**97.1%**	*65.4%*
	Absent					
Frantic efforts to avoid abandonment		10.3%	*4.0%*	10.3%	*3.5%*	**53.8%**
	Present	89.7%	**96.0%**	89.7%	**96.5%**	*46.2%*
	Absent					
Paranoid ideation or dissociative		10.6%	*5.0%*	91.0%	7.6%	**44.2%**
symptoms		89.4%	**94.9%**	9%	92.4%	*55.8%*
	Present					
	Absent					
**SUD**						
Alcohol						
	Present	56.8%	*30.0%*	**77.9%**	61.4%	**73.1%**
	Absent	43.2%	**70.0%**	*22.1%*	38.6%	*26.9%*
Cannabis						
	Present	51.8%	*17.5%*	**87.6%**	49.1%	**76.9%**
	Absent	48.2%	**82.5%**	*12.4%*	50.9%	*23.1%*
Cocaine						
	Present	42.2%	*10.2%*	**80.0%**	*32.7%*	**76.9%**
	Absent	57.8%	**89.8%**	*20.0%*	**67.3%**	*23.1%*
Stimulants						
	Present	24.2%	*4.0%*	**54.5%**	*13.5%*	**44.2%**
	Absent	75.8%	**96.0%**	*45.5%*	**86.5%**	*55.8%*
Sedatives						
	Present	8.1%	*0.6%*	**19.3%**	*1.2%*	**25.0%**
	Absent	91.9%	**99.4%**	*80.7%*	**98.8%**	*75.0%*
Others						
	Present	30.7%	*3.4%*	**70.3%**	*18.1%*	**53.8%**
	Absent	69.3%	**96.6%**	*29.7%*	**81.9%**	*46.2%*
**Aggressiveness**						
Absent		39.0%	**70.0%**	*17.3%*	*30.4%*	*23.1%*
Minor aggressive behaviour		17.3%	13.0%	18.6%	18.7%	23.1%
Severe aggressive behaviour		43.7%	*17.0%*	**64.1%**	**50.9%**	53.8%
**Impulsivity**						
Motor						
	Middle - High	61.1%	*33.3%*	**91.0%**	59.6%	**76.9%**
	Low	38.9%	**66.7%**	*9%*	40.4%	*23.1%*
Attentional						
	High	11.2%	*2.8%*	**23.4%**	*3.5%*	**30.8%**
	Middle	49.6%	*28.3%*	**60.0%**	**59.1%**	61.5%
	Low	39.2%	**68.9%**	*16.6%*	37.4%	*7.7%*
Non planning						
	High	9.2%	*1.1%*	**18.6%**	6.5%	**19.2%**
	Middle	68.3%	*59.3%*	**75.2%**	71.9%	67.3%

Low	22.5%	**39.6%**	*6.2%*	21.6%	13.5%
**Self-harm or suicidal behaviour**					
Absent	71.4%	**88.1%**	*58.6%*	**81.9%**	*15.4%*
< 3	19.6%	*9.6%*	**33.1%**	*14.0%*	**34.6%**
≥ 3	9.0%	*2.3%*	8.3%	*4.1%*	**50.0%**

Characteristics **positively** associated with the profiles **in boldface (value test ≥ 2),**
Characteristics *negatively* associated with the profiles *in italics* (*value test* ≥ *2*).
